# Areal differences in the utilization of endovascular therapy for acute ischemic stroke

**DOI:** 10.1093/eurpub/ckaf154

**Published:** 2025-09-09

**Authors:** Ulla Junttola, Siiri Hietanen, Sanna Lahtinen, Juha-Matti Isokangas, Lasse Raatiniemi, Timo Kaakinen, Merja Vakkala, Janne Liisanantti

**Affiliations:** Research Group of Anesthesiology, Medical Research Center Oulu, Oulu University Hospital, Oulu, Finland; Department of Neurology, Oulu University Hospital, Oulu, Finland; Research Group of Anesthesiology, Medical Research Center Oulu, Oulu University Hospital, Oulu, Finland; Department of Internal Medicine, Oulu University Hospital, Oulu, Finland; Research Group of Anesthesiology, Medical Research Center Oulu, Oulu University Hospital, Oulu, Finland; Department of Anesthesiology, Oulu University Hospital, Oulu, Finland; Research Group of Anesthesiology, Medical Research Center Oulu, Oulu University Hospital, Oulu, Finland; Department of Radiology, Oulu University Hospital, Oulu, Finland; Research Group of Anesthesiology, Medical Research Center Oulu, Oulu University Hospital, Oulu, Finland; Division for Prehospital Emergency Medicine, HEMS Unit, Oulu University Hospital, Oulu, Finland; Research Group of Anesthesiology, Medical Research Center Oulu, Oulu University Hospital, Oulu, Finland; Department of Anesthesiology, Oulu University Hospital, Oulu, Finland; Research Group of Anesthesiology, Medical Research Center Oulu, Oulu University Hospital, Oulu, Finland; Department of Anesthesiology, Oulu University Hospital, Oulu, Finland; Research Group of Anesthesiology, Medical Research Center Oulu, Oulu University Hospital, Oulu, Finland; Department of Anesthesiology, Oulu University Hospital, Oulu, Finland

## Abstract

Endovascular therapy (EVT) is standard care for acute ischemic stroke due to large vessel occlusion, but its availability is limited in areas with long distances. It has also been demonstrated that there are differences in the utilization of thrombectomy related to socioeconomic factors. The aim of this study is to examine regional differences in the utilization of mechanical thrombectomy and outcome within one comprehensive stroke center district in terms of distance and income. This retrospective single-center study included 352 patients with mechanical thrombectomy in Oulu University Hospital catchment area between the years 2015 and 2019. Socioeconomic status was determined according to the income of residential area. Age-adjusted rate was significantly higher in the highest income areas compared to the lowest and middle third areas; 52.29 (95% CI, 42.85–61.72)/100 000 vs. 34.33 (95% CI, 28.14–40.52) vs. 38.03 (95% CI, 31.14–44.92)/100 000 inhabitants/year. The corresponding rates in rural areas were: 73.37 (95% CI, 53.23–93.51) vs. 37.11 (95% CI, 28.66–45.57) vs. 45.44 (95% CI, 34.72–56.16)/100 000 inhabitants/year. In this study, we found significant differences in the utilization of the EVT within one comprehensive stroke center district. These differences are explained by the income and the rurality of the residential area.

## Introduction

Endovascular therapy (EVT) is standard care for acute ischemic stroke resulting from a large vessel occlusion [1]. EVT should be provided within 24 hours from the symptom onset [2]. Early initiation of EVT after stroke is associated with improved outcomes and lower mortality [3, 4]. The availability of EVT is limited in areas with long distances, since EVT can only be performed at thrombectomy-capable comprehensive stroke centers (CSCs). It has also been demonstrated that there are differences in the utilization of thrombectomy related to socioeconomic factors [5, 6].

Previous studies report significant differences in the need of hospital care within hospital district of Northern Finland; lower socioeconomic status has been shown to be associated with increased need of healthcare resource usage despite the government funded healthcare system, that should be equal to all the citizens [7–9]. In the same region, the geographical factors have been associated with limited availability of care in patients with acute ischemic stroke requiring thrombolysis and patients with acute myocardial infarction [10, 11]. Weather conditions in Northern-Finland limit the use of HEMS transportation during autumn and winter seasons.

In previous studies, there has been differences in the access of thrombectomy within areas in terms of socioeconomic and geographical factors [5, 6]. The utilization of mechanical thrombectomy has been shown to be higher in areas with high population density [5]. Additionally, the rate of EVT increases with wealth in both insurance-based and tax-funded healthcare systems [5, 12]. Moreover, in sparsely populated areas the availability of acute neurological care is highly depending on the distance to the neurological unit [11]. We have previously demonstrated a high rate of complications and high short-term mortality associated with long distances and sparse population in patients admitted for mechanical thrombectomy in CSC providing care for population located in Northern Finland [13]. However, according to our knowledge, the number of studies focusing on this item in terms of socioeconomic factors and geographical factors are lacking. In this study, we aimed to examine regional differences in the utilization of mechanical thrombectomy and outcome within CSC district in terms of distance and income.

## Methods

This retrospective study was conducted in Oulu University Hospital, which serves as a CSC for the entire Northern Finland. The setting is tertiary academic hospital serving as a CSC for population of 738 690 inhabitants in 2018 with surface area of 172 900 km^2^, covering ∼50% of the surface area of Finland. There are four primary stroke centers (PSCs) in the CSC district area referring patients after initial stroke management, which includes intravenous thrombolysis and other medical management. The nearest PSC is located 100 km and the farthest 175 km from the CSC. During the study period emergency medical services (EMSs) were dispatched to stroke with high priority for suspected stroke when the symptom onset is less than 6 hours and in case of unknown time of symptom onset, up to 24 hours from last known well. Dispatch system is nationwide and criteria-based. In the study area, there are one paramedic and one physician-staffed helicopter emergency medical services (HEMS) unit. Physician-staffed-HEMS are dispatched to stroke only if requested by ground EMS. HEMS are used for secondary transports from PSC to CSC primarily in one of the four PSCs when the weather conditions enable the air transportation.

The study protocol was accepted by the hospital administration (reference number 268/2019). Following the local protocol and due to retrospective study design, no statement from the ethics committee was obtained.

### Patients

All patients living in the CSC district and admitted to Oulu University Hospital for EVT between the years 2015 and 2019 were included into the study. The data were extracted from the electronic medical records and consisted of patient demographics, data concerning the acute disease, procedural data including time from stroke onset to groin puncture and time from hospital admission to groin puncture and recorded complications. The data extraction is described in detail in our previous study [13]. Comorbidity was assessed by using Charlson Comorbidity Index (CCI). CCI is a scoring method, which categorizes patient’s comorbidities based on the diagnoses found in medical records [14]. Stroke severity was assessed by using the National Institutes of Health Stroke Scale (NIHSS). NIHSS is a neurological examination rating scale with higher scores indicating greater clinical deficit [15]. Disability was assessed by using the modified Rankin Scale, which is the most widely used outcome measure in stroke trials and ranges from 0 (no symptoms) to 6 (death) [16]. The dates of death of the deceased were retrieved from the hospital discharge registry.

### Residential areas

#### Income

Statistics Finland provides free database concerning demographic data of all the postal code areas in Finland. For the study, each postal code area represented in the study population were included. The postal codes of the home addresses of each patient in the study population were retrieved from the hospital′s patient management system and the data concerning the postal code area of home addresses was added from the database of Statistics Finland. Index year 2015 was selected to categorize the population. First the postal code areas were ranked from the lowest median annual net income of the inhabitants to the highest. Then the study population was divided into three categories according to this rank: lowest third according to residential area’s median annual net income 13 768–18 744 €, middle third 18 745–20 708 €, and the highest third 20 709–25 958 €.

#### Rurality

The postal code areas were divided to rural and urban according to population density; areas with population density <76.7 inhabitants/km^2^ were considered rural [17]. Straight line distance from the areas to the Oulu University Hospital was calculated using the coordinates provided by Statistics Finland. Population in the highest income areas was younger, lived more often in urban areas, had lower unemployment rate and was less often retired compared to low and middle third income areas. (Supplementary Table S1).

### Statistical analysis

Statistical analysis was performed using SPSS 27 for Windows. Categorical variables are presented in numbers (*n*) and percentages (%) and tested using Pearson’s Chi-Square. Continuous variables are presented in medians and 25th and 75th percentiles (25th–75th PCT). Distributions between groups were tested using non-parametric Kruskal–Wallis test. Rates for thrombectomy, complications, and mortality were calculated for the represented postal code areas and the age-adjustment was performed by weighting rates for each the age group of 5-year intervals. The rates are reported in events per 100 000 person years.

## Results

A total of 353 patients underwent EVT in Oulu University Hospital between the years 2015 and 2019. Their median age was 71 years (61–80) and 54.7% of them were male. There were no differences in the patient demographics between the populations in different income areas. (Table 1) The patients living in the highest income areas had shorter distance to the Oulu University Hospital and shorter time from stroke onset to EVT compared to patients living in lower income areas. There were no differences in EVT outcomes between the groups (Table 2).

**Table 1.** Patient characteristics at baseline

	Overall (*n* = 353)	Lowest income areas (*n = *117, 33.1%)	Middle-income areas (*n = *118, 33.4%)	Highest income areas (*n = *118, 33.4%)	*P* value
Age (years), median (IQR)	71 (61–80)	70 (58–81)	71 (65–79)	73 (62–79)	.648
Gender (male), *n* (%)	193 (54.7)	55 (47.0)	73 (61.9)	65 (55.1)	.074
Smoking, *n* (%)	60 (17.0)	20 (17.1)	16 (13.6)	24 (20.3)	.390
Residence in urban area, *n* (%)	159 (45.0)	48 (41.0)	44 (37.3)	67 (56.8)	.006
Prior stroke, *n* (%)	64 (18.1)	17 (14.5)	23 (19.5)	24 (20.3)	.471
CCI-score, median (IQR)^a^	4 (2–6)	4 (2–6)	4 (3–6)	4 (3–6)	.467
mRS 0–2 baseline, *n* (%)^b^	317 (91.4)	100 (87.0)	109 (93.2)	108 (93.9)	.135
NIHSS baseline, median (IQR)^c^	14 (9–18)	13 (10–17)	14 (9–18)	13 (6–16)	.051
Hospital district					
CSC, *n* (%)^d^	185 (52.4)	58 (49.6)	52 (44.1)	75 (63.6)	.620
PSC 1, *n* (%)^e^	36 (10.2)	11 (9.4)	16 (13.6)	9 (7.6)	
PSC 2, n (%)	54 (15.3)	26 (22.2)	16 (13.6)	12 (10.2)	
PSC 3, *n* (%)	53 (15.0)	8 (6.8)	27 (22.9)	18 (15.3)	
PSC 4, *n* (%)	25 (7.1)	14 (12.0)	7 (5.9)	4 (3.4)	

a CCI = Charlson comorbidity index.

b RS = modified Rankin scale of functional dependence ranges from 0 (no symptoms) to 6 (death).

c NIHSS = National Institutes of Health Stroke Scale range from 0 to 42, with higher scores indicating a greater clinical deficit.

d CSC = Comprehensive Stroke Center.

e PCS = Primary Stroke Center.

**Table 2.** Procedural data and outcome.

	Overall (*n = *353)	Lowest income areas (*n = *117, 33.1%)	Middle-income areas (*n = *118, 33.4%)	Highest income areas (*n = *118, 33.4%)	*P* value
IVT, *n* (%)^a^	170 (48.4)	56 (48.7)	53 (44.9)	61 (51.7)	.585
Distance from hospital (km), median (IQR)	111 (17–173)	124 (7–174)	138 (89–180)	36 (11–163)	<.001
Distance from hospital, rural areas (km), median (IQR)	141 (79–197)	152 (93–181)	131 (89–190)	121 (29–201)	<.273
Distance from hospital, urban areas (km), median (IQR)	15 (4–165)	5 (3–150)	153 (90–174)	11 (7–93)	<.001
Time from stroke onset to groin puncture (min), median (IQR)	312 (215–504)	319 (215–530)	327 (240–635)	263 (168–400)	.003
Time from stroke onset to groin puncture, rural areas (min), median (IQR)	313 (225–433)	325 (229–426)	305 (226–505)	320 (215–435)	.731
Time from stroke onset to groin puncture, urban areas (min), median (IQR)	300 (195–680)	315 (186–810)	405 (290–758)	255 (160–365)	.001
Time from hospital admission to groin puncture (min), median (IQR)	76 (46–118)	81 (50–135)	64 (43–102)	82 (48–115)	.042
Time from hospital admission to groin puncture, rural areas (min), median (IQR)	75 (46–117)	67 (46–135)	80 (50–105)	71 (40–115)	0.818
Time from hospital admission to groin puncture, urban areas (min), median (IQR)	80 (46–122)	90 (64–141)	55 (30–83)	90 (62–166)	0.001
NIHSS after EVT, median (IQR)^b^^,^^c^	8 (4–16)	7 (4–16)	8 (4–15)	9 (4–16)	0.903
Recanalization result					
TICI 3, *n* (%)^d^	186 (60.4)	69 (66.3)	56 (54.4)	61 (60.4)	
TICI 2B–2C, *n* (%)	105 (29.7)	33 (28.2)	41 (34.7)	31 (26.3)	
TICI 0–2A, *n* (%)	17 (4.8)	2 (1.7)	6 (5.1)	10 (8.5)	0.178^f^
Neurological complication, *n* (%)	69 (19.5)	22 (18.8)	20 (16.9)	27 (22.9)	0.508
Medical complication, *n* (%)	221 (62.6)	67 (57.3)	74 (62.7)	80 (67.8)	0.250
ICU admission, *n* (%)^e^	158 (44.8)	49 (41.9)	55 (46.6)	54 (45.8)	0.452^f^
Hospital LOS (days), median (IQR)	6 (4–8)	5 (3–8)	5 (3–7)	6 (4–8)	0.104
mRS after 90 days^g^					
0–2, *n* (%)	119 (38.0)	44 (41.5)	36 (37.1)	39 (35.5)	0.004
3–4, *n* (%)	94 (30.0)	28 (26.4)	32 (33.0)	34 (30.9)	
5, *n* (%)	37 (11.8)	18 (17.0)	6 (6.2)	13 (11.8)	
6, *n* (%)	63 (20.1)	16 (15.1)	23 (23.7)	24 (21.8)	

a IVT = intravenous thrombolysis.

b NIHSS = National Institutes of Health Stroke Scale range from 0 to 42, with higher scores indicating a greater clinical deficit.

c EVT = endovascular therapy;

d TICI = the modified thrombolysis in cerebral infarction scale, ranges from 0 to 3, with a grade of 2b, 2c, and 3 indicating successful reperfusion.

e ICU = intensive care unit.

f Fisher’s exact test.

g mRS = modified Rankin scale of functional dependence ranges from 0 (no symptoms) to 6 (death).

The overall age-adjusted utilization rate of EVT was 36.89 (95% CI, 33.04–40.74)/100 000 inhabitants/year. The age-adjusted rate in highest income areas was 52.29 (95% CI, 42.85–61.72)/100 000 inhabitants/year, which was 1.5-fold compared to middle income areas 34.33 (95% CI, 28.14–40.52)/100 000 inhabitants/year and 1.4-fold to lowest income areas 38.03 (95% CI, 31.14–44.92)/100 000 inhabitants/year. The highest rate was found in rural high-income areas 73.37 (95% CI, 53.23–93.51)/100 000 inhabitants/year, which was 2.3-fold compared to rural middle income areas 37.11 (95% CI, 28.66–45.57)/100 000 inhabitants/year. The age-adjusted rates in urban areas were comparable between the income areas. The age-adjusted rate of disability at 90 days was highest in highest income areas 28.84 (95% CI, 20.60–37.09)/100 000 inhabitants/year, which was three-fold compared to middle income areas 10.32 (95% CI, 7.04–13.61)/100 000 inhabitants/year. The overall age-adjusted 4-year mortality rate was 17.54 (95% CI, 14.12–20.96)/100 000 inhabitants/year. The highest 4-year mortality rate was found in the highest income areas 28.50 (95% CI, 18.78–38.23)/100 000 inhabitants/year (Table 3).

**Table 3.** Crude and age-adjusted rates of utilization of EVT/100 000 inhabitants/year between the income areas.

	Overall	Lowest income areas	Middle-income areas	Highest income areas
Crude rate	12.31 (11.03–13.60)	15.75 (12.90–18.60)	11.26 (9.23–13.29)	11.05 (9.06–13.04)
Age-adjusted rate	36.89 (33.04–40.74)	38.03 (31.14–44.92)	34.33 (28.14–40.52)	52.29 (42.85–61.72)
Crude rate rural areas	14.35 (12.33–16.37)	21.74 (16.61–26.87)	11.66 (9.01–14.32)	12.76 (9.26–16.26)
Age-adjusted rate rural areas	39.79 (34.19–45.39)	45.44 (34.72–56.16)	37.11 (28.66–45.57)	73.37 (53.23–93.51)
Crude rate urban areas	10.55 (8.91–12.19)	11.28 (8.09–14.47)	10.64 (7.50–13.79)	10.03 (7.62–12.43)
Age-adjusted rate urban areas	35.41 (29.91–40.91)	40.58 (29.10–52.06)	31.53 (22.21–40.85)	44.50 (33.84–55.15)
Crude rate of disability after 90 days (mRS 3–5)^a^	4.58 (3.80–5.37)	6.19 (4.40–7.98)	3.63 (2.47–4.78)	4.40 (3.14–5.66)
Age-adjusted rate of disability after 90 days (mRS 3–5)	14.94 (12.38–17.50)	17.65 (12.55–22.76)	10.32 (7.04–13.61)	28.84 (20.60–37.09)
Crude 4-year mortality rate	3.53 (2.84–4.22)	4.31 (2.81–5.80)	3.44 (2.31–4.56)	3.09 (2.04–4.14)
Age-adjusted 4-year mortality rate	17.54 (14.12–20.96)	23.32 (15.24–31.40)	17.08 (11.50–22.66)	28.50 (18.78–38.23)

a mRS = modified Rankin scale of functional dependence ranges from 0 (no symptoms) to 6 (death).

The age-adjusted utilization rate of thrombectomy was highest in PSC 1 district, which is the nearest and located 100 km from the CSC. The lowest rates were in PSC districts 2 and 4 (Table 4).

**Table 4.** Crude and age-adjusted rates of utilization of EVT and distance to CSC of different PCS.

	CSC^a^	PCS 1^b^	PCS 2	PCS3	PSC 4
Crude rate	12.26 (10.50–14.03)	17.69 (11.91–23.48)	12.03 (8.82–15.24)	11.09 (8.11–14.08)	11.34 (6.89–15.79)
Age-adjusted rate	42.70 (36.55–48.85)	50.46 (33.98–66.94)	33.87 (24.83–42.90)	42.42 (31.00–53.84)	26.94 (16.38–37.50)
Distance to CSC (km)	–	92.4	165.6	174.2	137.9

a CSC = Comprehensive Stroke Center.

b PCS = Primary Stroke Center.

## Discussion

The main finding of this study was that there are significant areal differences in the utilization of EVT for ischemic stroke. The present results are partly in line with previous studies showing increased utilization of EVT along with the increasing wealth [5, 6]. Attenello *et al.* [5] demonstrated that EVT is performed more frequently among patients with better socioeconomic status living in higher income neighborhoods. Furthermore, Brinjikji *et al.* [6] found that socioeconomic disparities in the utilization of EVT exist even when the patients were treated in CSC. In Finland, as well as in the vast majority of other European countries, the government- or tax-funded healthcare systems offers equal access for all citizens to acute stroke treatments. Contrary to our results, a French study reported no differences in the access to reperfusion therapy according to socioeconomic status (SES) [18]. However, similarly to our results, a Danish study found that lower SES was associated with lower rates of both intravenous thrombolysis and EVT. In their study, prehospital delay was considered an important factor mediating the socioeconomic disparities in the utilization of reperfusion therapies [12]. The present cohort was admitted to the hospital within a large hospital district in Northern Finland, and it is obvious that long distances have an impact on prehospital delay and the utilization of EVT.

We found significant differences in the utilization of EVT in rural areas while in urban areas the age-adjusted rates were comparable. Rural area patients have been found to be more hesitant to seek help and contact the EMS compared to urban area patients, despite similar self-awareness of stroke symptoms [19]. Another important factor mediating differences among rural area patients might be living alone, which is associated with diminished rates of recanalization therapies among acute ischemic stroke patients [20].

Contrary to a previous study, we report that highest income area patients were more likely to be functionally dependent at 90 days [21]. The mechanisms of how SES influences functional outcomes remain unclear. Early and intensive rehabilitation system play a critical role in the recovery process after stroke [21, 22]. Higher rate of disability among high-income area patients may reflect delayed and ineffective rehabilitation system of these areas. The patients from the high-income areas had significantly shorter distance and shorter time from stroke onset to EVT. The high rate of EVT may reflect on poor outcome. However, we did not find any major differences between the income groups concerning prestroke functional status, comorbidity or baseline stroke severity, indicating that patient populations from different income areas were comparable. The only exception was a trend towards older age in patients living in high-income areas, which did not reach statistical significancy.

The higher rate of EVT in high-income areas reflects partly the distances, but also public awareness; the age-adjusted rate for EVT in rural areas was two-fold in high-income areas compared to lower income areas. When compared to other types of healthcare resource usage within the same region, the present results differ significantly [7–9]. According to previous study, EMS missions were significantly more common in low-income areas as well as non-transport decisions [9]. The lower rate in the utilization of EVT may be explained by under triaging but also the lack of awareness of the stroke symptoms, which may have an impact on the EMS dispatches.

### Limitations

This study has several limitations. First, the design is retrospective, limiting the availability of the data. However, registry-based study design allowed us to gain higher number of patients representing equally the whole hospital district compared to prospective study design with informed consent. Second, we did not have the data of those patients who had large vessel occlusion but did not receive EVT due to time delays. Accordingly, we did not have data of suspected stroke patients without indication for EVT. Especially including the patients receiving intravenous thrombolysis but not EVT could have enhanced the study. Moreover, we were not able to include EMS data or data from emergency central to analyze the impact of triaging to the results. Unfortunately, the Finnish legislation does not allow us to combine patients within areas of different types of healthcare providers, including the regional PSCs and EMS. Third, dividing the study population according to the median annual income of the residential area instead of personal income can be considered a limitation, but there might be value in residential area-based approach, when planning the use of healthcare resources or preventive measures.

It is notable that the presented distances are straight-line measurements and therefore they do not reflect the actual travel time from the place of the residence to the CSC.

### Clinical impact

The lower rate of EVT in low income and rural areas may reflect the availability of the treatment but also the public awareness. According to the present results, the stroke treatment chain still needs to be developed. The development includes adequate availability of EMS in rural areas. Additionally, patients with clinical symptoms referring to the large vessel occlusion should be recognized accurately and transported directly from the scene to CSC. However, this cannot be implemented to the disadvantage of patients who benefit the most from rapid intravenous thrombolysis administration in the nearest PSC. Furthermore, the utilization of HEMS in stroke could be improved with better dispatch accuracy and landing sites in hospitals. In Finland, HEMS is traditionally seldom used for the patient transportation compared to the HEMS, e.g. in Denmark and Norway [23]. Non-transport decisions of the patients with neurological symptoms are rare in Northern Finland [24, 25], indicating that these patients are well recognized by EMS.

## Conclusion

In this study, we found significant differences in the utilization of the EVT within one CSC district. These differences are explained by the income and the rurality of the residential area.

## Supplementary Material

ckaf154_Supplementary_Data
