# Supplementary material for: Areal differences in the utilization of endovascular therapy for acute ischemic stroke
Source: Eur J Public Health. 2025 Sep 9;35(6):1087–91. doi: 10.1093/eurpub/ckaf154 (PMC12707464; doi:10.1093/eurpub/ckaf154)
Supplement: ckaf154_Supplementary_Data [file ckaf154_supplementary_data.docx]

Supplementary Table S1: Demographic characteristics of the postal code areas

|  | Overall  (n=171) | Lowest income areas  (n=59) | Middle income areas  (n=57) | Highest income areas  (n=55) | P-value |
| --- | --- | --- | --- | --- | --- |
| Inhabitants, n | 571 721 | 148 609 | 208 962 | 214 150 |  |
| Population density (inhabitants/km^2^), median (IQR) | 12.6  (3.3.-203.2) | 3.6  (1.1-151.3) | 10.1  (3.6-209.7) | 63.8  (13.4-242.4) | <0.001 |
| Age (years),  median (IQR) | 42 (39-46) | 46 (41-50) | 43 (40-45) | 38 (35-41) | <0.001 |
| Inhabitants in urban areas, n (%) | 301 445 (52.7) | 85 079 (57.3) | 82 701 (39.6) | 133 613 (62.4) | <0.001 |
| Working age population, n (%) | 261 666 (45.8) | 66 213 (44.6) | 93 294 (44.6) | 102 159 (47.7) | <0.001 |
| Unemployed, n (%) | 41 503 (15.9) | 13 213 (20.0) | 14 747 (15.8) | 13 543 (13.3) | <0.001 |
| Working, n (%) | 220 052 (84.1) | 52 889 (80.0) | 78 547 (84.2) | 88 616 (86.7) | <0.001 |
| Retired, n (%) | 143 050 (25.0) | 44 797 (30.1) | 58 215 (27.9) | 40 038 (18.7) | <0.001 |
